# NIPBL and STAG1 enable loop extrusion by providing differential DNA–cohesin affinity

**DOI:** 10.1073/pnas.2514190122

**Published:** 2025-08-05

**Authors:** Raman van Wee, Roi Asor, Yiwen Li, David Drechsel, Mariia Popova, Gabriele Litos, Iain F. Davidson, Jan-Michael Peters, Philipp Kukura

**Affiliations:** ^a^Physical and Theoretical Chemistry, Department of Chemistry, Kavli Institute for Nanoscience Discovery, Dorothy Crowfoot Hodgkin Building, University of Oxford, Oxford OX1 3QU, United Kingdom; ^b^Department of Physiology, Anatomy and Genetics, University of Oxford, Oxford OX1 3QX, United Kingdom; ^c^Research Institute of Molecular Pathology, Vienna BioCenter, Vienna 1030, Austria

**Keywords:** Cohesin, biomolecular mechanism, protein–protein interactions, mass photometry, single molecule

## Abstract

DNA loop extrusion by cohesin has emerged as a critical pathway for chromosome organization. In vitro single-molecule experiments indicate that loop extrusion requires the assembly of a heteropentameric complex consisting of the SMC1/SMC3 heterodimer, STAG1, NIPBL, and the kleisin SCC1. The complexity of the complete extrusion machinery, consisting of multiple subunits, DNA binding sites, and ATPases poses substantial challenges for revealing the underlying biomolecular mechanism. As a result, a number of different models have been proposed, many of which do not agree on key mechanistic aspects, such as the details of DNA loading, holoenzyme assembly, or the consequences of ATP binding and hydrolysis. Here, we use mass photometry to comprehensively quantify all the key biomolecular interactions required for DNA loop extrusion. We find that STAG1 binds tightly to the trimeric complex formed by the SMC1/SMC3 heterodimer and SCC1, and together they weakly, but cooperatively, bind the DNA. Full-length NIPBL tightly binds DNA, acting as a DNA anchor during the mechanochemical loop extrusion cycle. Cohesin mutants incapable of head engagement, and those lacking DNA-binding domains in the ATPase heads show negligible differences in overall DNA-affinity, suggesting a minor role of these features for DNA binding. Instead, we find an ATP-modulated DNA binding site created by the interaction of STAG1 with SMC1/SMC3/SCC1, important for repeated grabbing and release of DNA critical to extrusion. Our results call for a careful reexamination of the proposed mechanisms and set energetic boundaries for future proposals.

## Significance

Loop extrusion has emerged as a fundamental process to maintain chromosome structure and dynamics. The molecular heterogeneity and cooperative effects within an ATP-modulated, multicomponent biomolecular machine complicate quantification of the underlying molecular interactions. As a result, our current understanding is mostly based on studies that investigate individual structural snapshots or quantify individual interactions, insufficient to conclusively determine the molecular functions of the constituent proteins. Here, we use mass photometry to quantify all protein–protein interactions involved in the formation of the cohesin holoenzyme and its dynamic binding to DNA. Our results point to a model where the cohesin Trimer (SMC1/SMC3/SCC) is bound to STAG1 in solution and NIPBL binds to DNA. STAG1–cohesin then loads by interacting with DNA and NIPBL.

**Fig. 1.**
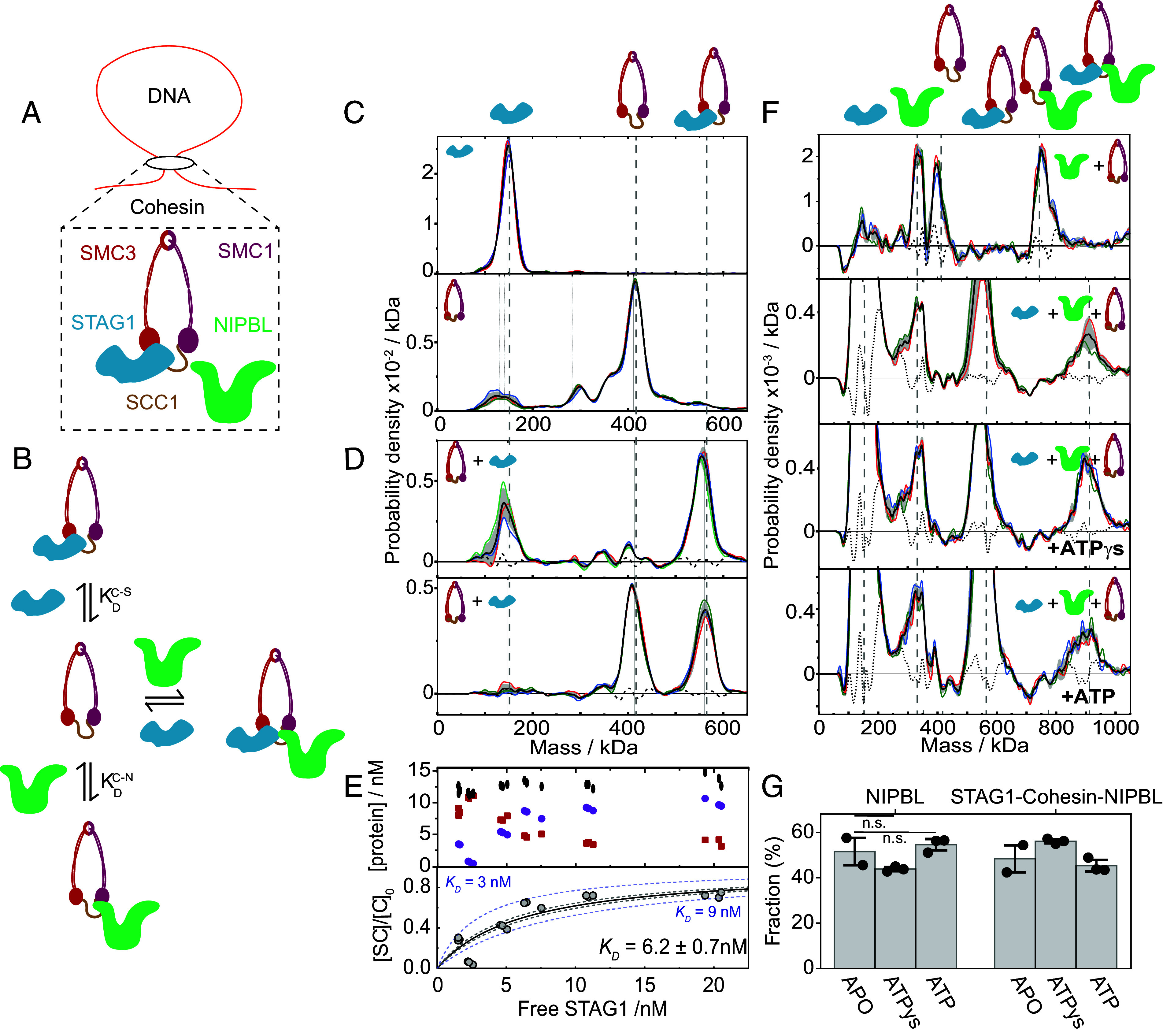
Quantification of the STAG1–cohesin affinity and demonstration of holoenzyme assembly without the need for DNA or ATP. (*A*) Schematic depiction of the cohesin holoenzyme. (*B*) Protein–protein interactions involved in the assembly of the holoenzyme. (*C*) Representative mass histograms of STAG1 (*Top*) and the cohesin Trimer (*Bottom*) with the position of the peak of interest indicated. (*D*) Representative mass histograms of the STAG1–cohesin interaction following the mixing of 150 nM STAG1 + 150 nM the cohesin Trimer (*Top*) and 80 nM STAG1 + 150 nM the cohesin Trimer (*Bottom*), all total protein concentration. (*E*) *Top*: quantification of the concentration of cohesin-STAG1 complex (purple circles), free cohesin Trimer (red squares), and total cohesin Trimer (black ellipses) for the STAG1 titration. *Bottom*: Hill plot, showing the molar fraction of the STAG1–cohesin complex as a function of the concentration of free STAG1 (*SI Appendix*, Fig. S5 for all distributions and fits). All mixtures were incubated at constant cohesin Trimer concentration which corresponds to 50 nM total protein concentration (12.6 ± 0.9 nM of effective cohesin Trimer concentration), and a STAG1 concentration ranging from 3 to 30 nM. Circles correspond to six different STAG1 concentrations with 3 technical repeats for each. Solid black curve corresponds to the best fitted Hill equation (*Materials and Methods*). Grey dashed curves represent the boundaries of the fitting error, and the dashed blue lines correspond to the expected Hill plot for the indicated *K*_D_ values and serve as visual guide for the scale of variation in the estimated dissociation constant. (*F*) Mass histograms following the subtraction of the residual signal, emphasizing the detected subunits and complexes of the cohesin holoenzyme (*SI Appendix*, Figs. S1 and S2). The subtracted mass histograms correspond to the equilibrated mixture of 150 nM of the cohesin Trimer sample mixed with 150 nM of: NIPBL (*Top*), STAG1 and NIPBL (second from the *Top*), and STAG1 and NIPBL in the presence of 2.5 mM ATP and ATPγS (as indicated). The increase in the peak width for the complete holoenzyme (905 kDa) may result from the natural decrease of the absolute mass resolution for high mass complexes owing to spatial variations in the roughness of the glass surface (15) as well as small variations in the measured interferometric contrast that arise from the large size of the holoenzyme and its conformational heterogeneity (15, 16) (*G*) Molar fractions of free NIPBL and NIPBL that is part of the NIPBL–cohesin-STAG1 complex out of the total NIPBL concentration for the mixtures with STAG1, NIPBL and the cohesin Trimer (*Bottom* three panels in *F*). Fractions were determined by considering the area under the two peaks. No significant difference is observed for the APO, +ATPγs, or +ATP case (*P* > 0.05 with a two-sided *t* test). Bars indicate the mean value across technical repeats (black dots) and black error bars represent the SD. All histograms indicate the expected masses of the components and complexes with vertical dashed grey lines and show the average (black curve), SD (grey error bars), and between 2 and 3 technical repeats (solid-colored curves). The error of the reconstruction of the experimental histogram from the histograms of the individual components is shown by the dashed black line (where applicable).

The interphase genome is organized into chromatin loops by cohesin (1–4), which is believed to function by binding to DNA and reeling flanking sequences into a loop that increases in size over time. This process is termed loop extrusion and has been visualized directly using single-molecule experiments (5, 6). Cohesin function relies on the formation of a holoenzyme consisting of the SMC1/SMC3 heterodimer, the largely intrinsically disordered kleisin SCC1 (also known as RAD21 or Mcd1), and the HAWKs (7) STAG1/STAG2/STAG3 (also known as Scc3), and NIPBL (also known as Scc2) (Fig. 1*A*) (8, 9). SMC1 and SMC3 form extended coiled-coil arms that dimerise at a “hinge” interface at one of their ends to adopt a V-shape. The other end of each arm contains an ATPase domain that is related to those found in ATP binding cassette (ABC) transporters (10), which are connected by SCC1, forming a ring-structure to which STAG1 and NIPBL bind (11). Mutations in cohesin subunits or its regulators are associated with several developmental diseases, collectively known as cohesinopathies (12–14).

Structural studies have revealed a variety of conformations at different stages of the ATPase cycle, including an open ring-shape, a closed rod-shape, and a bent state (17–22). Following these findings and a series of complementary studies, multiple models for loop extrusion have been proposed, including swing-and-clamp, ratchet, hold-and-feed, and reel-and-seal models (8, 9, 23, 24). These models not only differ in the conformational choreography driving loop extrusion but also in fundamental details controlling the key DNA–protein interactions. For example, the scrunching, hold-and-feed, and reel-and-seal models do not propose joint DNA binding by STAG1 and the cohesin Trimer, while the Brownian ratchet model does. Similarly, anchoring and grabbing functions have been assigned interchangeably to both STAG1 and NIPBL. Further, in the reel-and-seal model, DNA affinity is enhanced upon ATP binding due to head engagement, while in others, it is due to binding of the SMC1/SMC3 hinge or heads.

The emergence of these contradicting pictures can ultimately be traced back to a lack of quantitative knowledge of the interactions between the key biomolecules involved in loop extrusion and their dependence on ATP binding and hydrolysis. The 1:1 interaction strengths and molecular vicinities have been studied through electrophoretic shift assays (19), single-molecule fluorescence (5, 6, 25), and electron microscopy (26, 27). The multimeric nature of the extrusion machinery, however, demands a complete characterization of the full thermodynamic landscape of the interactions, which requires observation and quantification of the heterogeneity of the molecular species.

In this study, we take advantage of the molecular resolution of mass photometry (MP) to quantify all key interaction strengths, their dependence on ATP binding, and the effect of mutations in the DNA-binding sites and in the ATP head engagement site. Our findings are consistent with the STAG1–cohesin complex grabbing and releasing DNA in an ATP-dependent manner while NIPBL holds on to DNA, thereby leading to loop extrusion. The resulting model offers an explanation as to why in contrast to cytoskeletal motor proteins such as myosins and kinesins, cohesin and other structural maintenance of chromosomes (SMC) complexes evolved the additional complexity of accessory proteins, an aspect that has not been discussed in the majority of existing models (8, 9, 23). Our results set a thermodynamic framework against which existing and future proposals for the molecular mechanism of loop extrusion should be tested.

## Results

### STAG1 and the Cohesin Trimer Interact with Low Nanomolar Affinity.

MP identifies and quantifies different species by single-molecule mass measurement and counting in solution (28–30). Individually purified components characterized by MP exhibit distributions dominated by a peak at the expected molecular mass, although additional purification impurities are also visible (*SI Appendix*, Fig. S1). To characterize the interactions underlying holoenzyme assembly (Fig. 1 *A* and *B*), we mix and incubate different components prior to MP analysis, where interactions and their strengths are evidenced by the disappearance of constituent peaks and the concomitant appearance of peaks at the expected complex mass. To isolate the mass distribution of species participating in the interaction, we have applied a background subtraction procedure that removes residual impurities in our protein preparations that do not participate in the reaction from the histogram using a spectral decomposition (*SI Appendix*, Figs. S2 and S3). As an example, consider the interaction between human STAG1 (148 ± 2 kDa, μ ± SD, Fig. 1 *C*, *Upper*) and the SMC1/SMC3/SCC1 cohesin Trimer (414 ± 1 kDa, μ ± SD, Fig. 1 *C*, *Lower*), which is expected to be strong, given that the two can easily be copurified (31, 32).

When we incubate STAG1 and the cohesin Trimer in a slight excess of STAG1, we find a complete disappearance of free cohesin Trimer (Fig. 1 *D*, *Upper*). Conversely, using an excess of cohesin Trimer results in a complete loss of STAG1 (Fig. 1 *D*, *Lower*). Both results point towards a low nM affinity, which we can quantify by performing a suitable titration, while ensuring that the mixture was incubated for longer than the time required to equilibrate (Figs. 1*E* and *SI Appendix*, Fig. S4). Throughout the titration, the concentration decrease of free cohesin Trimer with increasing amounts of STAG1 is on par with the increase of STAG1- cohesin, and the total cohesin Trimer partial concentration remains constant (12.6 ± 0.9 nM, Fig. 1*E*). This, together with the fact that no new mass peaks appear throughout the titration (*SI Appendix*, Fig. S5), confirms that no other species than STAG1 and the cohesin Trimer are participating in the reaction. Any unidentified species can thus be assigned as background. A subsequent fit to the Hill equation yields *K*_d_ = 6.2 ± 0.7 nM in close agreement with the value obtained from evaluating individual mass distributions directly (33, 34) of *K*_d_ = 5.4 ± 1.7 nM (Figs. 1*E* and *SI Appendix*, Figs. S5 and S6). These results indicate that a titration as well as single-shot measurements with MP reveal the molecular affinities.

### The Cohesin Holoenzyme Assembles without DNA or ATP.

Having successfully tested our approach, we turn to the interaction between NIPBL and the cohesin Trimer. Using individual mass distributions, we find a *K*_d_ of 20 ± 4 nM for the interaction of NIPBL with the cohesin Trimer (Fig. 1 *F*, *Top* panel), in good agreement with ITC measurements of NIPBL and the kleisin subunit (20.4 nM) (35), whose interaction has also been observed in yeast (36). We found NIPBL to self-oligomerize and aggregate to some extent (*SI Appendix*, Fig. S7) in agreement with previous in vitro reports (19, 37) and in line with cohesin’s ability to form ordered clusters in yeast (38). Still, our measurements show that the most abundant state of the protein under our experimental conditions is its monomeric form.

Having shown strong interactions of the cohesin Trimer with NIPBL and with STAG1 individually, we are now in a position to probe the assembly of the complete, pentameric holoenzyme. Upon mixing all three proteins, the complete holoenzyme (905 kDa) assembled, with no observable difference in the relative abundances in the presence of ATP or ATPγs (Fig. 1 *F*, *Bottom* three panels, *G* and *SI Appendix*, Fig. S8). We confirmed that the holoenzyme hydrolyzes ATP and binds ATPγs with an ATPase assay (*SI Appendix*, Fig. S9), observing enhanced ATP hydrolysis in the presence of DNA in line with previous results (5, 39, 40). The observation that ATP does not affect the 1:1 interactions of STAG1 and NIPBL with the cohesin Trimer (*SI Appendix*, Figs. S10 and S11) supports the hypothesis that ATP binding or hydrolysis do not affect the effective affinities forming the holoenzyme. This observation is in line with the finding that STAG1 can be readily copurified with ATP binding or hydrolysis deficient mutant forms of the cohesin Trimer (39, 41, 42). We also did not detect a direct interaction between STAG1 and NIPBL (*SI Appendix*, Figs. S10 and S11), suggesting that the strong pairwise interactions with the cohesin Trimer drive the assembly of the holoenzyme. Overall, our results show that the holoenzyme is stable without DNA and ATP at low nanomolar concentrations.

### STAG1 and the Cohesin Trimer Bind DNA Cooperatively.

Loop extrusion requires binding of the holoenzyme to DNA. The cohesin holoenzyme can exist in a “clamp” conformation in which it simultaneously contacts DNA at sites on NIPBL, STAG1, and on top of the SMC1/SMC3 ATPase heads (26, 27, 43). Similar DNA binding sites, plus an additional one at the SMC1/SMC3 hinge, were identified using electrophoretic mobility shift assays and were shown to have high nanomolar to micromolar affinities (19). All five of these DNA binding sites are essential for loop extrusion (19). However, the contribution of each of these interactions to the recruitment of the cohesin holoenzyme to DNA is debated (44). Some reports ascribe this function primarily to NIPBL (6, 26, 27, 45–47), while others contradict this (24, 48, 49).

**Fig. 2.**
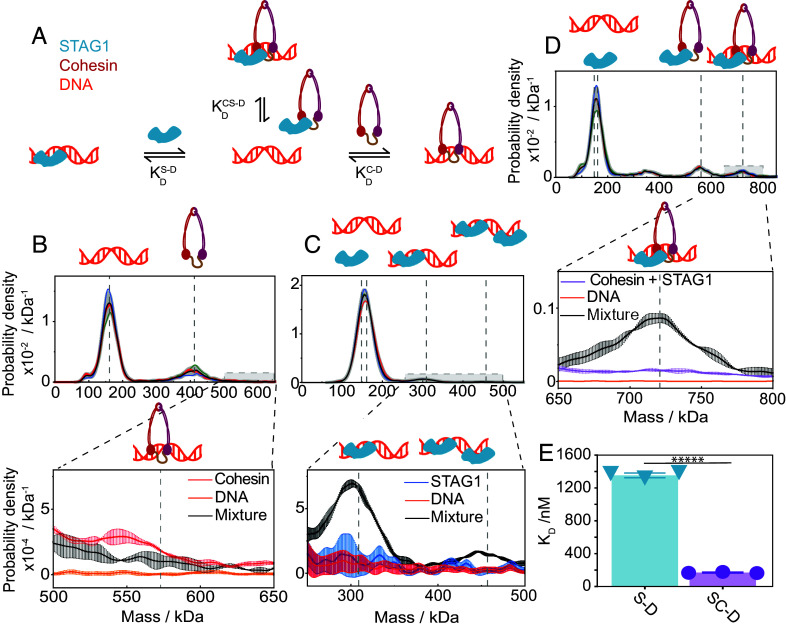
Cooperative interaction of STAG1–cohesin with DNA. (*A*) Illustration of possible interactions of STAG1, the cohesin Trimer and DNA with their dissociation constants indicated. (*B*–*D*) *Top*: Mass histograms of mixtures of 150 nM 302 bp minicircular dsDNA with 150 nM cohesin Trimer (*B*), 150 nM STAG1 (*C*), and 150 nM of the cohesin Trimer and STAG1 (*D*). Black curves and grey error bars correspond to the average mass histogram of three technical replicates and their SD. Individual mass histograms of the individual replicates are presented as colored curves. For all mass histograms, the expected masses of the relevant subunits (DNA, the cohesin Trimer and STAG1) and their complexes are indicated by the vertical dashed grey lines. *Bottom*: Mass histograms zoomed around the expected mass of the DNA–protein complexes. For all cases, black curves and error bars correspond to the measured mean distribution and SD of mixtures (*B*: the cohesin Trimer + DNA, *C*: STAG1 + DNA, and *D*: the cohesin Trimer + STAG1 + DNA). Colored curves and error bars correspond to the measured mass distribution of the individual components when measured alone. The ratio between the area beneath the mixtures curves (black) and the individual component curves (colored) represents our ability to detect and quantify the formation of the DNA–protein complex [0.54 ± 0.03 (*A*), 2.73 ± 0.11 and 3.08 ± 0.04 (*B*), and 11.2 ± 0.02 (*C*)]. Grey dashed vertical lines indicate the expected mass of the cohesin–Trimer–DNA complex (*B*), STAG1–DNA 1:1 and 2:1 complexes (*C*), and STAG1–cohesin–DNA complex (*D*). (*E*) Single-shot *K*_D_ values for the interactions of STAG1 and STAG1–cohesin with DNA, which are 1.35 ± 0.03 µM & 169 ± 6 nM, respectively. Bars indicate the mean value across technical repeats (black dots) and black error bars represent the SD. The *K*_D_ of the cohesin–Trimer–DNA interaction is beyond the detection limit of mass photometry. Assuming a conservative detection limit of 1 nM, we estimated the lower bound for the *K*_D_ to be >2 µM. Since the STAG1 and DNA are unresolvable, the total area under their mutual peak in (*C*) was assumed to contain 50% STAG1 and 50% DNA, variations in the extracted *K*_D_ values assuming different ratios are shown in *SI Appendix*, Fig. S13. *****P < 0.000005 with a one-sided *t* test.

To address this question, we measured the interaction of the cohesin Trimer and STAG1 with DNA (Fig. 2*A*). First, we compared binding to circular and linearized DNA constructs with the same sequence and found that purified STAG1–cohesin preferentially binds to the circular, supercoiled construct (*SI Appendix*, Fig. S12). Preferential binding to supercoiled and circular DNA over relaxed and linear DNA has also been observed for condensin and Smc5/6 (50–52), possibly because the energetic penalty for DNA deformation upon DNA binding has already been paid (53). Since cohesin can bind to DNA without entrapping it inside its ring structure (5, 54–56), it is possible that cohesin preferentially binds circular DNA for the same reasons. However, it is also possible that some spontaneous entrapment of DNA inside cohesin ring’s occurred under our assay conditions (57) and that this contributed to the preferential binding of circular over linear DNA. We used the circular construct in all subsequent experiments.

Upon mixing DNA and the cohesin Trimer at equimolar concentrations of 150 nM, we observed clear peaks at the expected masses of DNA and the cohesin Trimer (164 ± 4 and 414 ± 1 kDa, both mean ± SD), but not at the mass of a 1:1 complex (578 kDa) (Fig. 2 *B*, *Top*). When zooming in on the mass range of the DNA in complex with the cohesin Trimer, we found that the signal was indistinguishable to that of the DNA or the cohesin Trimer when measured individually (Fig. 2 *B*, *Bottom*). We therefore conclude that the interaction of the cohesin Trimer with DNA is too weak to be observed at the concentrations used and must be in the micromolar range or higher, in line with previous results based on fluorescence polarization (19, 58).

Given that the affinity of the cohesin Trimer for DNA is too low to associate with DNA at nuclear concentrations [(SCC1) = 330 nM] (59), either STAG1, NIPBL, or both must play a facilitating role. STAG1 is known to contain three DNA-binding patches (19, 58), and indeed when we incubated STAG1 with DNA, we observed 1:1 and 2:1 (STAG1:DNA) interactions (Fig. 2*C*). These results show that STAG1 by itself binds weakly to DNA with *K*_d_ = 1.35 ± 0.03 µM suggesting that STAG1 could facilitate binding of the cohesin Trimer to DNA.

We thus equilibrated STAG1 and the cohesin Trimer at equimolar concentrations of 150 nM and then added DNA. In these experiments, we did observe formation of a STAG1–cohesin–DNA complex (Fig. 2*D*). Comparing the STAG1 fraction bound to DNA (Fig. 2*C*) relative to the fraction of STAG1–cohesin on DNA (Fig. 2*D*) reveals that the latter is larger, suggesting a stronger interaction. Indeed, when we quantify the *K*_d_ from these measurements, we find a DNA affinity of 169 ± 6 nM for the STAG1–cohesin complex (Fig. 2*E* and *SI Appendix*, Figs. S10, S11, and S13). The 8-fold increase in affinity indicates a cooperative effect caused by simultaneous interaction of both STAG1 and the cohesin Trimer with DNA, an effect reproduced with different protein purification batches and DNA constructs (*SI Appendix*, Fig. S14). These results add to the body of evidence showing that NIPBL (or analogs) is not strictly required for binding the cohesin Trimer to DNA in vitro (25, 39, 60), although NIPBL is frequently referred to as “the cohesin loader” (26, 27, 35, 45, 46, 48, 61, 62).

### NIPBL Stabilizes the Cohesin Complex on DNA Independent of ATP.

Despite the cooperative effect of STAG1–cohesin formation on DNA binding, a high nM affinity would still be insufficient for DNA-binding at nuclear concentrations (59). This suggests the need for additional stabilization of the cohesin–DNA interaction. While STAG1 has been proposed to serve as a DNA-anchor (8, 19, 49), a similar role has been attributed to NIPBL in other studies (63).

**Fig. 3.**
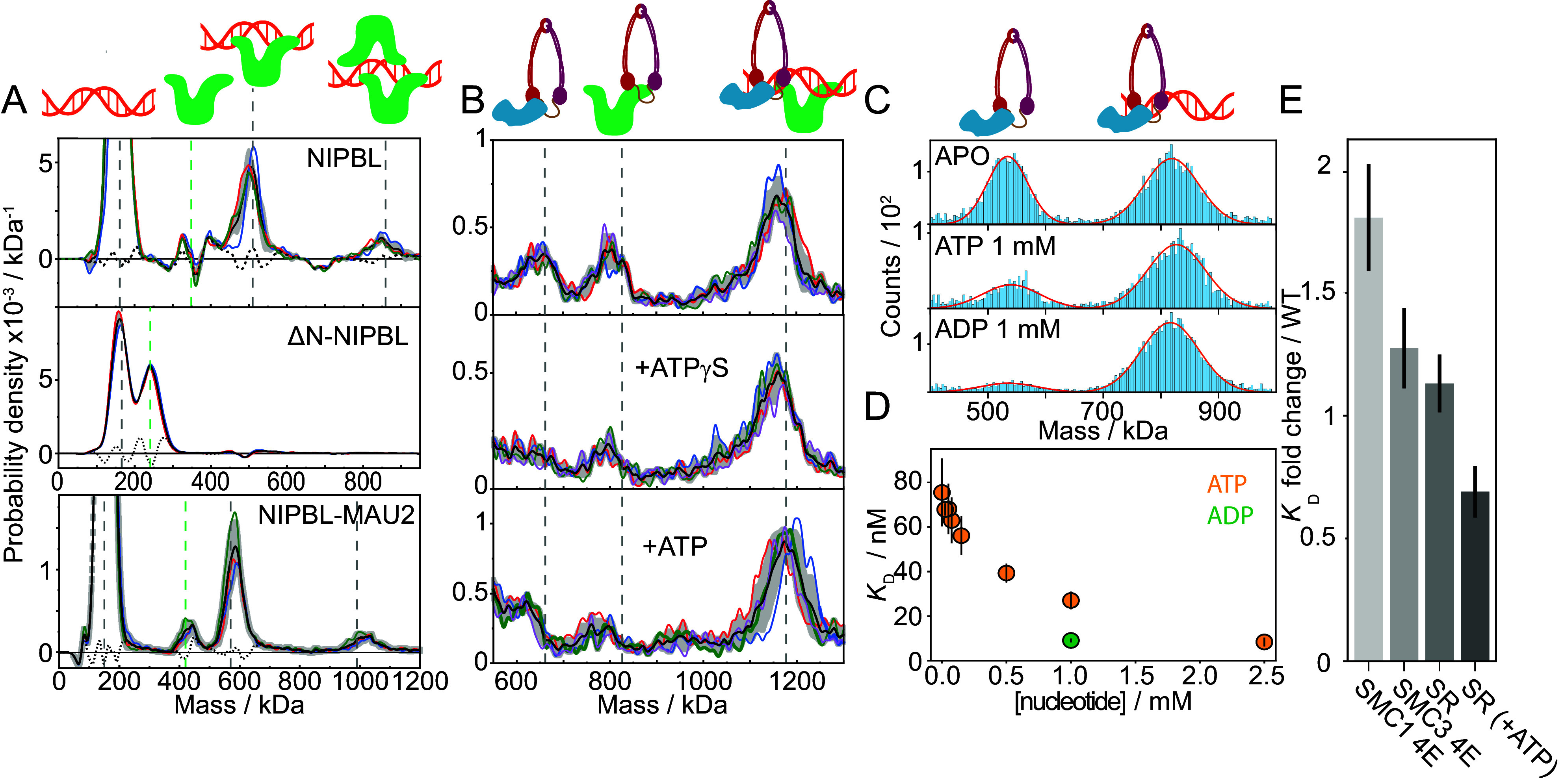
NIPBL stabilizes the holoenzyme on the DNA, while ATP binding modulates the interactions of STAG1–cohesin with DNA. (*A*) Residual subtracted mass distribution for a mixture of 150 nM 302 bp minicircle dsDNA with 150 nM NIPBL (*Top*), 150 nM ΔN–NIPBL (*Middle*), or NIPBL–MAU2 (*Bottom*). Average distribution, SD, and individual repeats are indicated by the black curve, grey error bars, and colored curves, respectively. Vertical lines indicating the expected masses of the different molecular species (NIPBL variants in green, DNA-containing species in grey). The expected mass of the NIPBL variants varies between the panels. (*B*) Mass distribution for mixing STAG1, the cohesin Trimer, NIPBL, and 302 bp minicircle dsDNA all at 150 nM (lay-out as in *A*). (*C*) Representative mass histograms and Gaussian fits of the mass region containing STAG1–cohesin (*Left* peak) and STAG1–cohesin–DNA (*Right* peak) under various nucleotide conditions. (*D*) Titration of ATP from 0 to 2.5 mM at 50 mM NaCl and the corresponding observed dissociation constant calculated by the relative abundances of the free STAG1–cohesin and STAG1-cohesin in complex with DNA. Yellow (green) circles and error bars correspond to the average and error of two technical replicates per ATP (ADP) concentration. (*E*) The averages and SD of the fold-change in the DNA affinity of STAG1–cohesin mutants relative to wildtype of at least three technical repeats per measurement (mutant and wildtype). Values are 1.81 ± 0.22 (SMC1 4E), 1.27 ± 0.16 (SMC3 4E), 1.13 ± 0.12 (SR/SR), and 0.69 ± 0.11 (SR/SR + ATP).

To resolve the contribution of NIPBL, we measured the DNA affinity of three commonly used variants: NIPBL, NIPBL–MAU2, and N-terminal truncated NIPBL (ΔN-NIPBL, NIPBL^1-21, 1041-2804^). Mixing equimolar concentrations of NIPBL and DNA (150 nM each) revealed peaks at 508 kDa and 851 kDa, indicative of 1:1 and 2:1 NIPBL:DNA interactions (Fig. 3*A* and *SI Appendix*, Fig. S15), as observed for STAG1–DNA (Fig. 2*C*). Since we did not detect free NIPBL in solution, we estimate the upper limit for the concentration of free NIPBL in solution to be 10 nM (prior to the 10-fold dilution during the data acquisition). Therefore, by considering the initial protein and DNA concentrations, we can set an upper limit to the dissociation constant to be approximately 10 nM. The NIPBL–MAU2 complex showed a similar affinity for DNA of 12.2 ± 0.2 nM in both the presence and absence of ATP (*SI Appendix*, Fig. S10), in line with previous reports showing that MAU2 does not have an intrinsic affinity for DNA (39). Our results show that among the cohesin Trimer, STAG1, and NIPBL, the latter has the highest DNA-affinity, and one that is sufficient for strong binding at nuclear concentrations [(NIPBL) = 200 nM] (59).

For the ΔN-NIPBL mutant on the other hand, which is often used due to its lower tendency for aggregation (5, 49) and employed in studies proposing STAG1 as a DNA anchor (49), we could not detect any complex formation with DNA following incubation at equimolar concentrations of 150 nM. This is indicative of a severely reduced DNA-affinity and a dissociation constant in the micromolar range or higher, as previously reported for this mutant (19, 49). These results suggest that the N terminus of NIPBL substantially contributes to DNA binding. Our results therefore show that truncating NIPBL’s N terminus results in a nonrepresentative interaction with DNA, unless the salt concentration is lowered to enhance the ΔN–NIPBL–DNA affinity (49).

When we mixed the three components of the holoenzyme (STAG1, the cohesin Trimer, and NIPBL) and then added DNA to the mixture, the intact holoenzyme associated with DNA (1,069 kDa, Fig. 3*B* and *SI Appendix*, Fig. S16), supporting the notion that ATP and MAU2 are not required for DNA binding in vitro (25, 31, 37, 39, 49, 60, 61). Moreover, we did not detect any unbound holoenzyme, which we did for the interaction between STAG1–cohesin and DNA (Fig. 2*D*), showing that the strong interaction of NIPBL with DNA increases the affinity of the holoenzyme for DNA. This enhancement was independent of ATP binding or hydrolysis, as the addition of ATPγs or ATP did not substantially alter the distribution.

### ATP Modulates the STAG1–Cohesin–DNA Affinity.

Successful loop extrusion requires one anchor point, and another that is modulated as the DNA is reeled in. Given the strength and ATP-independence of the NIPBL-DNA interaction, we examined the potential ATP-dependence of the STAG1–cohesin–DNA interaction. We repeated our DNA binding experiments in the presence of ATP, which is not hydrolyzed in the absence of NIPBL (5, 19, 39), and ADP. We found that the addition of either nucleotide increased the STAG1–cohesin affinity for DNA (Fig. 3*C*). A titration of ATP revealed a substantial affinity increase from 75 ± 15 nM (in APO) to 8 ± 2 nM (at 2.5 mM ATP) (Fig. 3*D*), in line with previous studies that have found a high micromolar to low millimolar affinity of subunits of the cohesin Trimer for ATP (40, 64) and an enhanced DNA affinity upon ATP binding (25, 62). For these experiments, STAG1–cohesin was purified as a single complex, rather than assembled in vitro, as in Figs. 1 and 2, which might explain the two-fold difference in affinity for the DNA relative to Fig. 2*E*.

As STAG1 does not have an ATP binding domain and does not bind DNA more tightly in the presence of nucleotides when measured alone (*SI Appendix*, Figs. S10 and S11), the affinity increase most likely stems from the cohesin Trimer. Indeed, we reproduced the same trend in a buffer of lower ionic strength in the absence of STAG1 (*SI Appendix*, Fig. S17). These results suggest that the structural rearrangement in the cohesin Trimer upon ATP binding (19, 26, 65, 66) increases the DNA affinity. Interestingly, we observed a tight interaction between the STAG1–cohesin complex with DNA in the presence of ADP, despite ADP being unable to engage the ATP heads (40).

Since most interactions of the holoenzyme with DNA are mediated by electrostatic forces with the phosphate backbone (26, 27, 43), the apparent affinity for DNA is expected to depend heavily on the ionic strength of the solution. Indeed, the interaction of STAG1–cohesin with DNA is modulated by the delicate interplay between ionic strength (decreasing affinity) (25, 49) and nucleotide presence (increasing affinity) (*SI Appendix*, Fig. S17). Above a threshold ionic strength, STAG1–cohesin is released and cannot rebind the DNA upon addition of ATP. When characterizing this interaction close to this ionic strength threshold one cannot easily decouple the effect from ATP binding from the effect of the increased ionic strength of the buffer, since ATP by itself, a multivalent ion, contributes substantially to the total ionic strength, leading to the dissociation of the complex upon ATP addition at 2.5 mM (*SI Appendix*, Fig. S18).

Finally, to dissect the contributions of the three previously revealed DNA binding sites on cohesin (19) on DNA affinity, we investigated cohesin variants with a single-point mutation in one of the DNA binding sites on the ATPase heads (SMC1 4E and SMC3 4E). When comparing the affinity of STAG1–cohesin to DNA of the mutants relative to wildtype, we did not detect substantial affinity changes for mutations in either DNA binding site on the ATPase head (Fig. 3*E*). Moreover, the SR/SR variant, which cannot engage the ATPase heads, had a similar DNA affinity as wildtype in the APO and ATP bound state. These results suggest that the individual DNA binding sites on the ATP heads are not critical to the overall DNA affinity and that head engagement after ATP binding (26) does not significantly enhance the affinity, hinting at a crucial contribution of the binding site on the hinge of the cohesin Trimer.

## Discussion

Our results show that the affinities of the cohesin Trimer with both STAG1 and NIPBL are in the low nanomolar range and independent of ATP, while a pairwise interaction between STAG1 and NIPBL was not observed. The high STAG1–cohesin affinity suggests that the proteins form a tight complex with a slow off-rate, which agrees well with previous observations that the two proteins coelute during purification (32, 67). The interaction of the cohesin Trimer with NIPBL is ~3 times weaker than STAG1 and is therefore expected to be more dynamic (5, 49, 68).

**Fig. 4.**
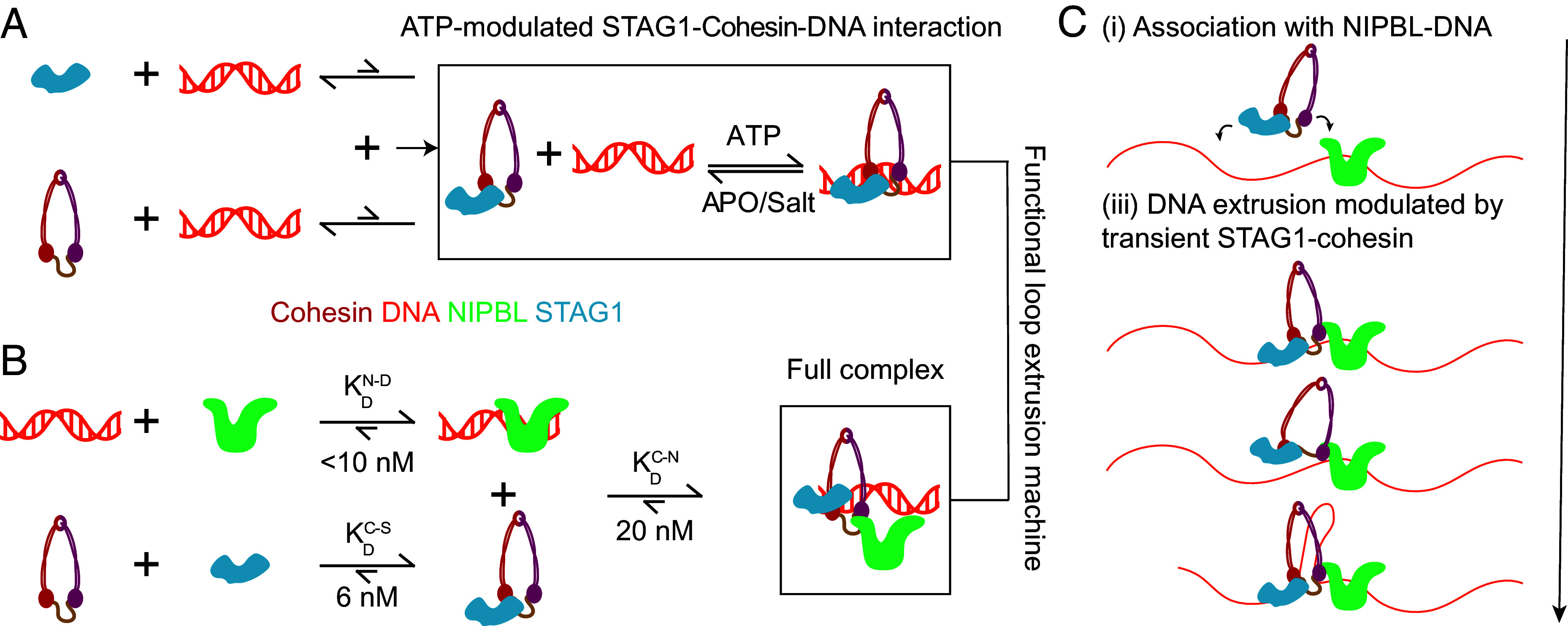
Quantitative description of the fundamental interactions governing cohesin holoenzyme–DNA affinity and their implications on loop extrusion. (*A* and *B*) Interaction strengths resulting in assembly of an ATP-modulated STAG1–cohesin–DNA interaction and the full complex. The relative lengths of arrows indicate the equilibrium of the reaction. (*C*) Proposed model for loop extrusion, based on characterization of the interaction strengths in (*A*) and (*B*) and the existing literature.

In contrast to the pairwise protein–protein interactions, the multivalent interaction landscape of cohesin subunits with DNA is more complex. These binding sites cooperatively interact with DNA with an apparent affinity that is modulated by ATP and changes in ionic strength. Our results reveal distinct roles for STAG1 and NIPBL in facilitating DNA–protein interactions during cohesin’s mechanochemical cycle (Fig. 4 *A* and *B*). Our finding of a cooperatively enhanced STAG1–cohesin–DNA interaction (Fig. 4*A*) suggests that STAG1 serves as a “DNA affinity enhancer” for the cohesin Trimer, such that the latter can grab, and hold on to DNA. This hypothesis is supported by the fact that STAG1 is not required for cohesin’s ATPase activity (5, 42), does not strongly affect the extent of DNA supercoiling induced by loop extrusion (69), and is required for loop extrusion (5) in buffer solutions with increased salt concentrations only (49).

Our results further suggest that the STAG1–cohesin complex is in principle able to bind DNA, but not at physiological salt concentrations. That cohesin variants with mutations in the DNA binding sites on the heads or deficient in head engagement do not exhibit a substantially decreased DNA affinity supports the hypothesis that the cooperative interaction of STAG1–cohesin with DNA and the affinity enhancement upon ATP binding rely on the hinge binding site on the cohesin Trimer. Further work will be required to understand how structural changes, such as coiled coil bending or kleisin gate opening, modulate the interaction with DNA. The weakening of the STAG1–cohesin–DNA interaction upon ADP release suggests that under the conditions required for DNA extrusion, STAG1 cannot establish a sufficiently strong connection to DNA to serve as an “anchor” in the extrusion process.

Out of the three tested holoenzyme subunits, NIPBL exhibited the highest affinity to DNA. While the interaction of NIPBL and the cohesin Trimer (20 nM) is weaker than that of STAG1 with the cohesin Trimer, it is sufficient to facilitate assembly of the complete holoenzyme on DNA. Our results suggest that in addition to its effect on cohesin’s ATPase activity (5, 39, 42), NIPBL is not strictly required for loading of the cohesin Trimer to DNA, but stabilizes the complete complex on DNA, in line with previous work that observed stabilization on chromatin (48) and on DNA curtains (70).

It is important to note that a high affinity and dynamic nature of the interaction are not mutually exclusive. Since the 1:1 interaction between NIPBL and DNA is electrostatic in nature and not sequence specific (71, 72), the long-range electrostatic attractions are expected to result in a relatively fast association rate. If we assume that the association rate is on the higher end for reported electrostatic interactions ($10^{8}-10^{9}\;M^{-1}s^{-1}$) (73) and given the upper limit of the dissociation constant of 10 nM for NIPBL–DNA interaction determined in this work, the dissociation rate constant would be 0.1 to 1 s^−1^. This corresponds to an average lifetime of 1 to 10 s (or longer when assuming slower association rates). We expect the lifetime of the complete complex to be longer, as direct interactions of STAG1 and the cohesin Trimer with DNA will contribute to the stabilization of the pentameric complex. Given typical in vitro loop extrusion rates of 0.5 to 2 kb/s (5, 6) and loop sizes of 33 kb (6), the expected lifetime of the functional complex is tens of seconds, which matches our calculation above and the experimentally observed lifetime of ~50 s for NIPBL and STAG1–cohesin–NIPBL (5, 68). Truncation of the N terminus of NIPBL severely reduces the affinity for DNA, underscoring the need to measure the effect of modifications to the protein sequence and explaining why a lifetime that is an order of magnitude lower was recently observed for this mutant (49).

Given nuclear concentrations of STAG1 (70 nM), the cohesin Trimer (330 nM for SCC1), and NIPBL (200 nM) (59), and the effective concentration of DNA binding sites being orders of magnitude larger, we predict that STAG1 and the cohesin Trimer exist as a complex, while NIPBL associates with DNA (because the DNA is much more abundant than the cohesin Trimer). The STAG1–cohesin complex is stabilized on DNA by binding naked DNA and a second interaction mediated by NIPBL (Fig. 4*C*). The complete holoenzyme binds DNA more tightly than any of the individual proteins, explaining why chromosome occupancy by NIPBL is reduced upon depleting cohesin (68, 74). Cohesin’s dynamic ATPase heads reel in DNA by undergoing consecutive ATP-dependent structural changes and affinity modulations, while NIPBL remains bound to DNA. Owing to the proximity to DNA, a second weak DNA-binding site on the cohesin Trimer prevents newly extruded DNA from slipping.

This “anchor” function of NIPBL agrees with the observation that PDS5, which is a binding partner of the cohesin release factor WAPL, competes with NIPBL for binding to kleisin (35, 42, 75–79). Moreover, the hypothesis that STAG1–cohesin predominantly interacts with NIPBL on DNA, and not in solution, is consistent with data in *Xenopus* showing that most soluble cohesin complexes do not contain NIPBL (80, 81) and that some chromatin remodelers have been reported to recruit NIPBL to chromatin (82). Furthermore, since it is known that CTCF barriers do not get extruded, but accumulate at the base of the loop, (83) it seems plausible that CTCF acts on the dynamic part of the extruder, which it encounters first. In our model, this would be STAG1, and indeed, a direct interaction between STAG2 and CTCF has been shown to affect the location of the extruded loop on the DNA (84).

In summary, we have quantified the biomolecular affinities of the cohesin holoenzyme, its interaction with DNA and the modulation by ATP and ADP, which have implications for the likely molecular choreography. Our data reveal a complex interaction landscape where affinities span at least three orders of magnitude in strength and are differentially modulated by ATP in a way that is key to the overall function of the cohesin machinery. Our study rationalizes the increased evolutionary complexity introduced by incorporating NIPBL and STAG1 accessory proteins, compared to other molecular motors, such as myosin and kinesin, as it allows the local modulation of binding affinities that facilitate loop extrusion as opposed to processivity. The development of assays to directly quantify the molecular composition of a loop extruding cohesin complex will test our proposed model, while providing rich insight into the interaction dynamics. More broadly, the fundamental approach based on MP presented here, in which all the interactions in the presence of substrate (here DNA) and fuel (here ATP) are quantified will prove useful in a wide range of mechanistic studies of biomolecular function and regulation.

## Materials and Methods

An extended *SI Appendix*, *Material and Methods* is provided, and includes the following: the expression and purification of all proteins used in this study, the synthesis of the MiniCircle DNA constructs, the mass photometry data acquisition and movie processing, histogram generation and partial concentrations calculations, histogram subtraction procedure and spectra reconstruction, procedure to fit the experimental histograms to quantify the partial concentrations, presentation for the subtracted histograms, experimental method for quantifying the protein–protein, the protein-DNA interactions, and the effect of ionic strength and nucleotides on these interactions, and the ATPase assay.

## Supplementary Material

Appendix 01 (PDF)

## Data Availability

.mp files containing landing assay recordings have been deposited in Oxford Research Archives (https://doi.org/10.5287/ora-mvyo6jpyj) (85). All study data are included in the article and/or *SI Appendix*.
